# A Candidate Gene Association Study of Bone Mineral Density in an Iranian Population

**DOI:** 10.3389/fendo.2016.00141

**Published:** 2016-10-27

**Authors:** Seyed Alireza Dastgheib, Alison Gartland, Seyed Mohammad Bagher Tabei, Gholamhossein Ranjbar Omrani, Marion Dawn Teare

**Affiliations:** ^1^Department of Medical Genetics, Shiraz University of Medical Sciences, Shiraz, Iran; ^2^Academic Unit of Bone Biology, Department of Oncology and Metabolism, The Mellanby Centre for Bone Research, The University of Sheffield, Sheffield, UK; ^3^Transgenic Technology Research Center, Shiraz University of Medical Sciences, Shiraz, Iran; ^4^Endocrine and Metabolic Research Center, Namazi Hospital, Shiraz University of Medical Sciences, Shiraz, Iran; ^5^Design Trials and Statistics, School of Health and Related Research (ScHARR), The University of Sheffield, Sheffield, UK

**Keywords:** VDR, P2RX7, candidate gene association, BMD, genetic epidemiology, Iranian population

## Abstract

The genetic epidemiology of variation in bone mineral density (BMD) and osteoporosis is not well studied in Iranian populations and needs more research. We report a candidate gene association study of BMD variation in a healthy cross-sectional study of 501 males and females sampled from the Iranian Multi-Centre Osteoporosis Study, Shiraz, Iran. We selected to study the association with 21 single nucleotide polymorphisms (SNPs) located in the 7 candidate genes *LRP5, RANK, RANKL, OPG, P2RX7, VDR*, and *ESR1*. BMD was measured at the three sites L2–L4, neck of femur, and total hip. Association between BMD and each SNP was assessed using multiple linear regression assuming an allele dose (additive effect) on BMD (adjusted for age and sex). Statistically significant (at the unadjusted 5% level) associations were seen with seven SNPs in five of the candidate genes. Two SNPs showed statistically significant association with more than one BMD site. Significant association was seen between BMD at all the three sites with the *VDR* SNP rs731246 (L2–L4 *p* = 0.038; neck of femur *p* = 0.001; and total hip *p* < 0.001). The T allele was consistently associated with lower BMD than the C allele. Significant association was also seen for the *P2RX7* SNP rs3751143, where the G allele was consistently associated with lower BMD than the T allele (L2–L4 *p* = 0.069; neck of femur *p* = 0.024; and total hip *p* = 0.045).

## Introduction

Osteoporosis is a skeletal disorder characterized by a reduction in the strength of bone with a consequent increase in bone fragility and susceptibility to fracture. Low bone mass is associated with micro-architectural deterioration of bone tissue (1). The loss of strength and bone mass can be as a result of a multitude of factors including the inability to reach optimal peak bone mass in early adulthood, increased resorption of bone having reached peak mass, or dysfunction of bone formation during bone remodeling (2). In this regard, hip fractures are the most important complication associated with osteoporosis and are linked to morbidity and mortality. While the cause of death is often due to other chronic disease, 25% of deaths are directly linked to the hip fracture or complications arising from the fracture such as pressure sores, infection, and bronchopneumonia (3). The 12-month survival rate post-hip fracture in the UK is 63% for men and 75% for women. In addition, one in two women and one in five men who are aged over 50 years will have suffered from an osteoporotic fracture in their remaining lifetime (4).

It is expected that by 2050, the world population will increase; as a result, it is also thought that the population of elderly people will grow (4). In the UK, by the year 2020, the over 85 years old age group is estimated to rise to 2.1 million, whereas this population was 1.2 million in 2002 (5). This increase in the aging population in the UK is predicted to cause double the number of osteoporotic fractures over the next 50 years (6). On this subject, genetic factors are important in the pathogenesis of osteoporosis, with links to a reduction in bone mineral density (BMD) and an increased susceptibility to osteoporotic fractures (7). Although genetic effects on BMD are mediated by several genetic factors with mild influences, more studies are needed for determining the relation between genetic factors and bone loss (8). In this regard, genome-wide association studies (GWAS) and candidate gene association studies are the two robust approaches to investigate correlation between genetic variation and disease risk to identify genome regions or candidate genes that contribute to osteoporosis disease (9, 10). The data obtained from these two approaches are growing with some suggestion of ethnic differences in the genetic associations with BMD (11).

The genetic epidemiology of variation in BMD is not well studied in Iranian populations and needs more research. To this end, we set out to examine the relative contributions of seven major candidate genes to BMD variation in a healthy cross-sectional study of males and females sampled from Shiraz, Iran.

## Materials and Methods

### Study Participants and Phenotypes

Subjects for the study were selected from The Shiraz Centre of the Iranian Multi-Centre Osteoporosis Study (IMOS), which is an ongoing study to determine reference values for bone densitometry in the female and male Iranian population from 2005. In the Shiraz Centre, 540 healthy subjects aged 20–75 years were selected randomly from 1080 subjects who were registered in the IMOS cohort and invited to participate in this study. Five hundred and one responded to the invitation and consented to participate (This sample of subjects is referred to as IMOS-AD throughout this report.). The study protocol was approved by the Research Ethics Committee of the Shiraz Medical University of the Iranian Ministry of Health and Medical Education. All the participants provided written informed consent after being fully informed of the nature of the study (in accordance with Helsinki Declaration).

Height was read from a free-standing height with the nearest 0.5 cm. Subsequently, subjects were weighed and body mass index (BMI) was calculated (kilograms per square meter).

### Identifying Candidate Genes

We aimed to study variants with established associations with BMD variation in other populations and/or those with relevance to the Iranian population. The genes with established associations were selected if evidence for association was reported and replicated through a candidate gene association study (CGAS), a meta-analysis of candidate gene association study (MA-C), a GWAS, and a meta-analysis of genome-wide association study (MA-G). Six key publications were used to select the genes with well-replicated associations with BMD (12–17). This led to the selection of *LRP5, RANK, RANKL, OPG*, and *ESR1* as these associations were reported by CGAS, MA-C, GWAS, and MA-G studies. These genes are also involved in three biological pathways, the Wnt/-catenin signaling pathway, the estrogen endocrine pathway, and the RANKL/RANK/OPG pathway. The *VDR* gene was also selected as it is the candidate gene related to osteoporosis most studied in Iranian populations (18, 19). *P2RX7* was also selected as it was a novel candidate gene (at the time of recruiting the subjects to the study) associated with BMD and osteoporotic fracture risk (20, 21).

### SNP Selection

Genome-wide association studies literature review was used for the strategic selection of single nucleotide polymorphisms (SNPs) that were the most relevant in BMD. The catalog of published GWAS in the National Human Genome Research Institute (NHGRI) was used to select associated SNPs for the selected candidate genes (22). This resulted in a total of 22 SNPs for the candidate gene association analysis with BMD variation in the IMOS-AD study. The specific SNPs selected were rs599083 (*LRP5*), rs3736228 (*LRP5*), rs884205 (*RANK*), rs3018362 (*RANK*), rs9594738 (*RANKL*), rs9594759 (*RANKL*), rs1021188 (*RANKL*), rs6469804 (*OPG*), rs4355801 (*OPG*), rs2062375 (*OPG*), rs2062377 (*OPG*), rs11995824 (*OPG*), rs2504063 (*ESR1*), rs1999805 (*ESR1*), rs4870044 (*ESR1*), rs1038304 (*ESR1*), rs2941740 (*ESR1*), rs6929137 (*ESR1*), rs3751143 (*P2RX7*), rs28360457 (*P2XR7*), rs731236 (*VDR*), and rs2228570 (*VDR*).

### Bone Mineral Density

Bone mineral densitometry was performed by dual-energy X-ray absorptiometry (DEXA) method (GE Lunar DPX-IQ Bone densitometer, GE Healthcare, Madison, WI, USA). It was used to measure appendicular and axial sites and measurements are reported in units of milligrams per square centimeter. Minimal radiation exposure was performed as produced in the method by Blake and Fogelman (23). Definitive data of BMD measurements of the total proximal femur and lumbar vertebrae (L2–L4) in the anterior–posterior position were obtained.

### Blood Sampling and DNA Extraction

Blood sampling was performed by the brachial vein puncture method (24, 25), and 200 μl of peripheral blood was collected from each participant in an EDTA tube. Finally, there were 501 participants whose blood samples and BMD were available.

The collected peripheral blood samples were subjected to DNA extraction through application of QIAamp 96 DNA Blood Kit. Finally, the extracted DNA samples were assessed by spectrophotometric measurements. The quality and purity of DNA was checked using a NanoDrop-1000 spectrophometer (Thermo Scientific) with an OD 260/280 ratio between 1.8 and 2 used as the criteria for acceptable DNA purity.

### Genotyping

Genotyping of the selected SNPs was performed by Competitive Allele-Specific Polymerase chain reaction (KASPar) assay (KBioscience services; United Kingdom). The KASPar assay SNP genotyping system from KBioscience is based on fluorescent resonance energy transfer (FRET) technology. Following completion of the reaction, the data reported were evaluated using the KlusterCaller 1.1 software (KBioscience). Hardy–Weinberg equilibrium analysis was conducted for quality control. One SNP (rs9594759) gave a *p*-value below the threshold of 0.001 (26) and was excluded from further analysis, so final results are presented for 21 SNPs only. All included SNPs had a call rate in excess of 95%.

All assays were tested on LGC Genomics’ in-house validation DNA prior to being run on the study samples. Assays were deemed to be working successfully when clusters were distinct and call rates consistently high. The data are automatically quality control checked on a per SNP basis. No template controls (NTCs) were included on each plate to enable the detection of contamination or non-specific amplification – these samples must not amplify during the reaction.

The number of genotypes that were callable must be greater than 90% and minor allele frequency should be greater than 2% unless the SNP is known to be a very low frequency.

### Statistical Analysis

GraphPad Prism 6 and IBM^®^ SPSS^®^ were used to analyze the data. The comparison of means for two independent groups was analyzed using the Student’s *t*-test. The *t*-test was used for all two group comparisons as the sample size in each group was always in excess of 50; the variables were approximately normally distributed, and sample SDs were similar. The association between BMD and the number of copies of the minor allele was analyzed using linear regression with the BMD variable as the outcome adjusting for the covariates age and sex of participant. For each linear regression analysis, the SNP genotype was coded as 0, 1, or 2 in terms of how many copies of the minor allele they carried. We decided to adjust for these two common confounders as this adjustment has been done in other studies (15) and adjusting for more covariates would reduce the statistical power. The adjusted association is reported along with 95% confidence intervals (95% CI). All reported *p*-values are nominal and not adjusted for multiple testing.

The power of the patient sample size of 501 to detect true associations depends upon the frequency of the genotypes. If the frequency of the common homozygote and the heterozygote were similar (each around 40%), then the sample would have 90% power to detect a true association responsible for a difference of 0.23 of a SD in BMD. If the heterozygote is as rare as 16% and the homozygote has frequency of 84%, then 501 patients would have 90% power to detect a difference in BMD of 0.4 of a SD. These calculations assume a two-sided 5% type 1 error rate.

## Results

### The BMI of the Males and Females within a Random Selection of Subjects from an Iranian Population

Each subject was supplied with a questionnaire from which demographic variables for this study including age, height, weight, and BMI were determined. The data were analyzed separately by gender as BMI is known to be higher in females than in males in the Iranian population (27). Menopausal status was recorded on the women, and 111 of the 248 women were postmenopausal. Statistical differences between genders were formally compared using a Student’s *t*-test. Statistical analysis showed that there were no statistically significant differences in age between the male and female subjects of our sample group, but the male subjects within our sample population had significantly higher weight and height metrics compared to the female subjects (*p* < 0.001). The calculated BMI was higher for females than for the male subjects within the selected Iranian population sample (*p* < 0.001) (Table 1).

**Table 1:** **Physical characteristics of both male and female study subjects**.

Statistical evaluation of the demographic characteristics of female and male subjects				
Variable	TotalMean (SD) Median (IQR)	Female Mean (SD) Median (IQR)	Male Mean (SD) Median (IQR)	*p*-Value
Age (years)	47.5 (16.0)	46.7 (15.7)	48.3 (16.3)	0.19
	47.0 (35.0–59.5)	47.0 (34.0–59.0)	47.0 (37.0–60.0)	
Height (cm)	161.2 (9.6)	154.5 (5.8)	168.4 (7.4)	<0.001
	160.0 (154.0–168)	155.0 (151.0–159.0)	168.0 (163.0–172.0)	
Weight (kg)	64.5 (11.8)	62.2 (11.8)	66.9 (11.4)	<0.001
	64.0 (56.0–72.0)	62.0 (54.0–70.3)	66.5 (58.0–73.0)	
BMI (kg/m^2^)	24.8 (4.4)	26.1 (4.6)	23.6 (3.67)	<0.001
	24.5 (21.5–27.6)	26.0 (22.3–29.6)	23.3 (20.1–25.9)	

*The age, height, weight, and BMI of females and males were summarized and compared. The male subjects had lower BMI values compared to female subjects. N = 501, Group specific means are compared using Student’s t-test. IQR, interquartile range*.

### The BMD of the Males and Females within a Random Selection of Subjects from an Iranian Population

The L2–L4 (L2–L4_BMD), neck of femur (Neck_BMD), and total hip BMD (Total_BMD) of the IMOS-AD sample consisting of 501 individuals was assessed using the DXA Scanner. Data from the whole sample population were separated into male and female groups to examine any gender associations with BMD. The gender-specific group means were compared using a Student’s *t*-test.

Analysis of the Iranian sample population showed that the male subjects had statistically significantly higher BMD means associated with L2–L4_BMD, neck_BMD, and total_BMD compared to that of the female population (*p* < 0.001) (Table 2).

**Table 2:** **Summary of BMD measurements in participants**.

BMD of participants				
Variable	Total Mean (SD) Median (IQR)	Female Mean (SD) Median (IQR)	Male Mean (SD) Median (IQR)	*p*-Value
L2–L4_BMD	1.031 (0.17)	1.001 (0.17)	1.060 (0.15)	<0.001
	1.030 (0.93–1.14)	1.030 (0.89–1.12)	1.031 (0.94–1.17)	
Neck_BMD	0.870 (0.15)	0.827 (0.14)	0.918 (0.14)	<0.001
	0.862 (0.77–0.96)	0.821 (0.75–0.92)	0.903 (0.81–1.00)	
Total_BMD	0.906 (0.15)	0.864 (0.15)	0.952 (0.13)	<0.001
	0.904 (0.82–1.00)	0.876 (0.79–0.95)	0.938 (0.85–1.04)	

*All BMD measurements are reported in mg/cm^2^. The female subjects had lower BMD values compared to male subjects. N = 501, Student’s unpaired t-test*.

### Analysis of Genotyping Data of 21 SNPS in Seven Selected Candidate Genes and BMD in the Iranian Subjects

*LRP5, RANK, RANKL, ESR1, VDR, P2XR7*, and *OPG* were selected following the literature review of genes for consideration in an Iranian population. The genetic association analysis was conducted assuming an additive allelic effect on the outcome variable BMD. The analysis was repeated for each BMD variable and for each SNP (i.e., 3 × 21 linear regression analyses). Regression analyses were performed adjusting for age and sex. The results in Table 3 show the linear regression coefficient for the association between the BMD outcome and the number of copies of the risk allele.

**Table 3:** **A summary of association between BMD and 21 selected SNPs**.

Summary of analysis by SNP and BMD site			
SNP (gene)	Minor allele	Per allele (95% confidence interval)	*p*-Value
rs599083 (LRP5)	G		
L2–L4		−0.013 (−0.032 to 0.006)	0.177
Neck		0.001 (−0.014 to 0.016)	0.868
Total hip		0.003 (−0.012 to 0.018)	0.732

rs3736228 (LRP5)	T		
L2–L4		−0.026 (−0.057 to 0.005)	0.103
Neck		0.008 (−0.016 to 0.033)	0.503
Total hip		0.014 (−0.011 to 0.039)	0.269

rs884205 (RANK)	A		
L2–L4		−0.009 (−0.034 to 0.015)	0.464
Neck		−0.016 (−0.036 to 0.003)	0.101
Total hip		−0.020 (−0.040 to −0.001)	0.042

Rs3018362 (RANK)	A		
L2–L4		−0.002 (−0.024 to 0.019)	0.846
Neck		−0.004 (−0.021 to 0.013)	0.655
Total hip		−0.005 (−0.022 to 0.012)	0.555

rs9594738 (RANKL)	T		
L2–L4		0.025 (0.004 to 0.047)	0.019
Neck		0.010 (−0.007 to 0.027)	0.236
Total hip		0.010 (−0.006 to 0.027)	0.225

rs1021188 (RANKL)	C		
L2–L4		−0.019 (−0.046 to 0.008)	0.161
Neck		−0.012 (−0.034 to 0.009)	0.261
Total hip		−0.018 (−0.040 to 0.003)	0.095

rs250463 (ESR1)	A		
L2–L4		0.003 (−0.017 to 0.022)	0.795
Neck		−0.001 (−0.020 to 0.014)	0.860
Total hip		−0.006 (−0.021 to 0.010)	0.465

rs1999805 (ESR1)	A		
L2–L4		−0.005 (−0.023 to 0.014)	0.628
Neck		−0.002 (−0.016 to 0.013)	0.819
Total hip		0.004 (−0.010 to 0.019)	0.567

Rs4870044 (ESR1)	T		
L2–L4		−0.001 (−0.020 to 0.021)	0.935
Neck		0.001 (−0.015 to 0.017)	0.897
Total hip		−0.000 (−0.016 to 0.016)	0.967

Rs1038304 (*ESR1*)	G		
L2–L4		0.015 (−0.005 to 0.036)	0.144
Neck		0.012 (−0.004 to 0.028)	0.141
Total hip		0.016 (−0.001 to 0.032)	0.062

Rs2941740 (*ESR1*)	G		
L2–L4		0.013 (−0.007 to 0.034)	0.203
Neck		0.060 (−0.014 to 0.019)	0.747
Total Hip		0.006 (−0.010 to 0.023)	0.448

Rs6929137 (*ESR1*)	A		
L2–L4		0.020 (−0.000 to 0.041)	0.054
Neck		0.007 (−0.000 to 0.024)	0.363
Total hip		0.015 (−0.002 to 0.031)	0.077

Rs7311236 (*VDR*)	C		
L2–L4		0.022 (0.001 to 0.043)	0.038
Neck		0.027 (0.011 to 0.044)	0.001
Total hip		0.029 (0.013 to 0.046)	< 0.001

Rs2228570 (*VDR*)	C		
L2–L4		0.014 (−0.007 to 0.036)	0.195
Neck		0.010 (−0.007 to 0.027)	0.248
Total hip		0.001 (−0.016 to 0.018)	0.918

Rs3751143 (P2RX7)	G		
L2–L4		−0.021 (−0.043 to 0.002)	0.069
Neck		−0.020 (−0.037 to −0.003)	0.024
Total hip		−0.018 (−0.035 to 0.000)	0.045

Rs28360457 (*P2RX7*)	A		
L2–L4		0.088 (−0.209 to 0.386)	0.559
Neck		0.078 (−0.156 to 0.312)	0.511
Total hip		0.073 (−0.164 to 0.310)	0.547

Rs6469804 (*OPG*)	A		
L2–L4		−0.023 (−0.043 to −0.003)	0.026
Neck		−0.011 (−0.027 to 0.005)	0.195
Total hip		−0.012 (−0.029 to 0.004)	0.129

Rs4355801 (*OPG*)	A		
L2–L4		−0.023 (−0.044 to −0.002)	0.029
Neck		−0.010 (−0.026 to 0.007)	0.240
Total hip		−0.013 (−0.029 to 0.003)	0.119

Rs2062375 (*OPG*)	G		
L2–L4		0.012 (0.008 to 0.032)	0.235
Neck		0.004 (−0.012 to 0.020)	0.638
Total hip		0.002 (−0.013 to 0.018)	0.761

Rs2062377 (*OPG*)	T		
L2–L4		0.017 (−0.004 to 0.037)	0.118
Neck		0.006 (−0.010 to 0.023)	0.452
Total hip		0.009 (−0.008 to 0.025)	0.301

Rs11995824 (*OPG*)	G		
L2–L4		−0.021 (−0.042 to −0.001)	0.044
Neck		−0.011 (−0.027 to 0.006)	0.199
Total hip		−0.013 (−0.029 to 0.005)	0.125

*The association between BMD and the number of copies of the minor allele was analyzed using multiple linear regression with the BMD variable as the outcome, adjusting for the covariates age and sex of participant. For each linear regression analysis, the SNP genotype was coded as 0, 1, or 2 in terms of how many copies of the minor allele they carried. To illustrate the interpretation, consider two people with the same gender and age but with genotypes TT and CC at the SNP Rs7311236. The results of this analysis would predict the total hip BMD of the person with CC to be 0.068 units higher than the person with genotype TT*.

Table 3 shows the association analyses grouped by SNP. Out of a total of 63 regression analyses of the data, 10 tests are statistically significant at the 5% level (three would be expected under the null hypotheses). However, the most notable result here is of the VDR polymorphism rs731236, which shows a statistically significant association at all the three BMD sites. The allele T is associated with lower BMD after adjusting for age and sex. The P2RX7 allele G for SNP rs3751143 is statistically significantly associated with lower BMD at two sites (the neck and total hip) and is marginally associated at the L2–L4 site.

## Discussion

This study looked at whether the BMD of a random selection of subjects from an Iranian population was genetically associated with a selection of candidate genes known to be associated with BMD. A total of 21 SNPs in 7 selected genes were evaluated for this study. The descriptive analysis of our random selection of subjects from the Iranian population showed that there were no significant differences between the mean ages of the males and females within the group. Additionally, the weight and height of the males were higher than that of the females, as observed in similar subject groups of other similar Iranian studies (27). These data are an indication that as the demographic characteristics of the randomly selected sample group was comparable to samples within other studies; further genetic studies would also be comparable to the literature. It is of interest to note that these data showed that male subjects had a lower BMI range compared to the female subjects and these data were consistent with findings from Bahrami et al. (27).

Data collected from the BMD analysis of our Iranian population samples showed that the total hip BMD of the male subjects was higher than the female subjects. The average BMD of our male sample group was 0.95 mg/cm^2^, and the females had an average BMD value of 0.86 mg/cm^2^. These data were consistent with BMD data obtained from other Iranian groups (28, 29), European groups (30) and American groups (31). These data suggest that the BMD pattern observed within our sample group was comparable to that of others used in similar genetic association studies (32, 33).

As mentioned previously, a low BMD is associated with the risk of developing osteoporosis (34). The relationship between BMD and BMI, including its weight and height constituents, is widely reported (35–37). In the literature, BMD is reported to be lower in lean postmenopausal women in the majority of studies. However, some studies have also reported increased BMD in lean postmenopausal women (38).

It is expected that many different genetic variants contribute to the regulation of BMD. Most of the variants are assumed to have small effect size, but there is evidence that rare variants of large effect size also contribute in some individuals (39). In the past 10 years, there have been advances in the identification and validation of osteoporosis susceptibility loci *via* large-scale association studies, meta-analyses, and GWAS of SNPs. A thorough review of latter developments has revealed that more than 15 genes can be assigned as confirmed BMD genes linked to osteoporosis (40).

The *LRP5, RANK, RANKL, P2RX7, ESR1, OPG*, and *VDR* genes selected for this study are known to have important roles in four well-defined biological pathways; the estrogen endocrine pathway, the Wnt/β-catenin signaling pathway, the RANKL/RANK/OPG pathway, and the vitamin D synthesis pathway (40). There are limited data related to effective genetic variants in osteoporosis, particularly in respect to the mentioned genes, in the Iranian population. The association analysis found 10 of the 63 tests resulted in *p* < 0.05 (Table 3). Under the null of no genetic association, we would only expect to see three of the results statistically significant. Hence, we do see some confirmation of associations with this set of genetic loci in the Iranian sample. However, the SNPs selected for analysis in this cohort are already established as risk SNPs, so in this evaluation, we are mainly interested to know if the overall pattern of association is similar to other populations or may be different. Such differences may be due to different population, alleles, or the impact of a distinct environment; hence, it is more the estimate and 95% CI that is of interest.

Out of the 21 selected SNPs, our genotyping data identified statistically significant associations between BMD and 7 of these SNPs: Rs731236, Rs3751143, Rs884205, Rs9594738, Rs4355801, rs6469804, and Rs11995824.

For the *RANK* gene, the present study showed that SNP rs884205 was associated with total hip BMD, and the same association was observed in studies by Styrkarsdottir et al. (30) within a European and East Asian population (15, 41). The present study further showed that *RANKL* SNP rs9594738 was also closely linked to spine BMD, similar to studies by Duncan et al. and Styrkarsdottir et al. in the European population (16, 42). The *OPG* SNPs rs2073804, rs1995824, and rs4355801 associations with spine BMD determined in the present study were also observed in other studies specific to spine and hip BMD in post/premenopausal women and the European population, respectively (33, 43). Although there are few published data on *P2RX7*, one study did correlate with ours in that SNP rs3751143 was associated with BMD in a similar number of subjects (44).

The most interesting result in our study is the association with the VDR SNP rs731246 (also known as *Taq*I), which is statistically significant over all the three measured BMD sites. Early work from Keen et al. (45) and Spector et al. (46) using UK Twin cohorts, as well as the work from Ferrari et al. (47), led to the identification of common polymorphisms in the VDR as being associated with BMD and an important target for the treatment of osteoporosis. However, there have been conflicting results (48), and more importantly meta-analyses including this genetic variant have not found consistent evidence for its association with BMD variation or osteoporotic fracture risk (8, 49–51). However, one Iranian study has shown a statistically significant association with the VDR SNP rs2228570 and BMD in females, though they did not examine the SNP rs731246.

While this study is a substantial contribution to the genetic epidemiology of variation in BMD in the Iranian population, it does have limitations. The sample size is modest, and this means the study is powered to detect larger effects but limits the potential for adjustment for additional covariates (such as menopausal status and BMI, height or weight). Over-adjustment for covariates impacts statistical power. When studying the association between the SNP genotypes and BMD, it is possible that for some SNPs, there was an imbalance of post- and premenopausal women in the genotype groups and this might lead to a spurious association. However, including age and gender as covariates would mitigate this to some extent. This study had no information on incidence of fractures hence we cannot be confident any of these associations would extend to increased facture risk.

In conclusion, our data demonstrate that the overall pattern of association of these established risk SNPs in the Iranian population are similar to those observed in other populations. From our data, we concur that BMD is genetically dependent (52), and that the VDR is statistically significantly associated with BMD in the Iranian population. Future prospective studies using this cohort would enable us to analyze fracture incidence, as well as the functional effects of these SNPs in bone cells.

## Author Contributions

SD designed and carried out the full IMOS-AD research project, and collected the data and patient samples. He conducted the statistical analysis and drafted the research paper. MT, AG, ST, and GO advised supervised and guided the design and analysis of the research project. All four were involved in drafting and revising the manuscript.

## Conflict of Interest Statement

The authors declare that the research was conducted in the absence of any commercial or financial relationships that could be construed as a potential conflict of interest.
